# Data on fundus images for vessels segmentation, detection of hypertensive retinopathy, diabetic retinopathy and papilledema

**DOI:** 10.1016/j.dib.2020.105282

**Published:** 2020-02-24

**Authors:** Muhammad Usman Akram, Shahzad Akbar, Taimur Hassan, Sajid Gul Khawaja, Ubaidullah Yasin, Imran Basit

**Affiliations:** a Department of Computer and Software Engineering, National University of Sciences and Technology, Islamabad, Pakistan; b Department of Information Technology, Bahauddin Zakariya University, Multan, Pakistan; c Armed Forces Institute of Ophthalmology, Rawalpindi, Pakistan

**Keywords:** Fundus images, Optic nerve head, Cotton wool spots, Hard exudates, Vessels segmentation

## Abstract

This paper presents a dataset that contains 100 high quality fundus images which are acquired from Armed Forces Institute of Ophthalmology (AFIO), Rawalpindi Pakistan. The dataset has been marked by four expert ophthalmologists to aid clinicians and researchers in screening hypertensive retinopathy, diabetic retinopathy and papilledema cases. Moreover, it contains highly detailed annotations for retinal blood vascular patterns, arteries and veins to calculate arteriovenous ratio (AVR), optic nerve head (ONH) region and other retinal anomalies such as hard exudates and cotton wool spots etc. The dataset is extremely useful for the researchers who are working in the ophthalmic image analysis.

**Table:** Specifications Table

Subject	Ophthalmology
Specific subject area	Human Retina, Hypertensive Retinopathy, Diabetic Retinopathy, Papilledema
Type of data	Images Excel file
How data were acquired	Images were acquired using TOPCON TRC-NW8 system
Data format	1. JPG (.jpg) raw Images of resolution of 1504x1000. 2. JPG (.jpg) Manually Annotated Images 3. Annotated values in EXCEL file (.xlsx)
Parameters for data collection	AVR, ONH, cotton wool spots and hard exudates annotations
Description of data collection	Fundus images of human eye, annotation is done by ophthalmologists.
Data source location	The data under investigation was obtained from Armed Forces Institute of Ophthalmology (AFIO). Rawalpindi, Pakistan 33.5962° N, 73.0450° E
Data accessibility	**Repository name:** Data on Fundus Images for Vessels Segmentation, Detection of Hypertensive Retinopathy, Diabetic Retinopathy and Papilledema **Data identification number:**https://doi.org/10.17632/3csr652p9y.1 **Direct URL to data:**https://data.mendeley.com/datasets/3csr652p9y/1
Related research article	Shahzad Akbar, Muhammad Usman Akram, Muhammad Sharif, Anam Tariq, Shoab Ahmed Khan, “Decision support system for detection of hypertensive retinopathy using arteriovenous ratio”, Artificial Intelligence in Medicine, Aug 1st, 2018, 90, pp. 15–24.

**Table:** 

**Value of the Data** • This data is publicly available and a benchmark for vessel segmentation, detection of hypertensive retinopathy, diabetic retinopathy and papilledema. • The provided data is useful for retinal blood vessels segmentation, artery/vein classification for computation of AVR, analysis of ONH and detection of various retinal abnormalities such as hard exudates and cotton wool spots. • The data is valuable for the development of automated techniques for the diagnosis of hypertensive retinopathy, diabetic retinopathy and papilledema.

## 1 Data

**Fig. 1:**
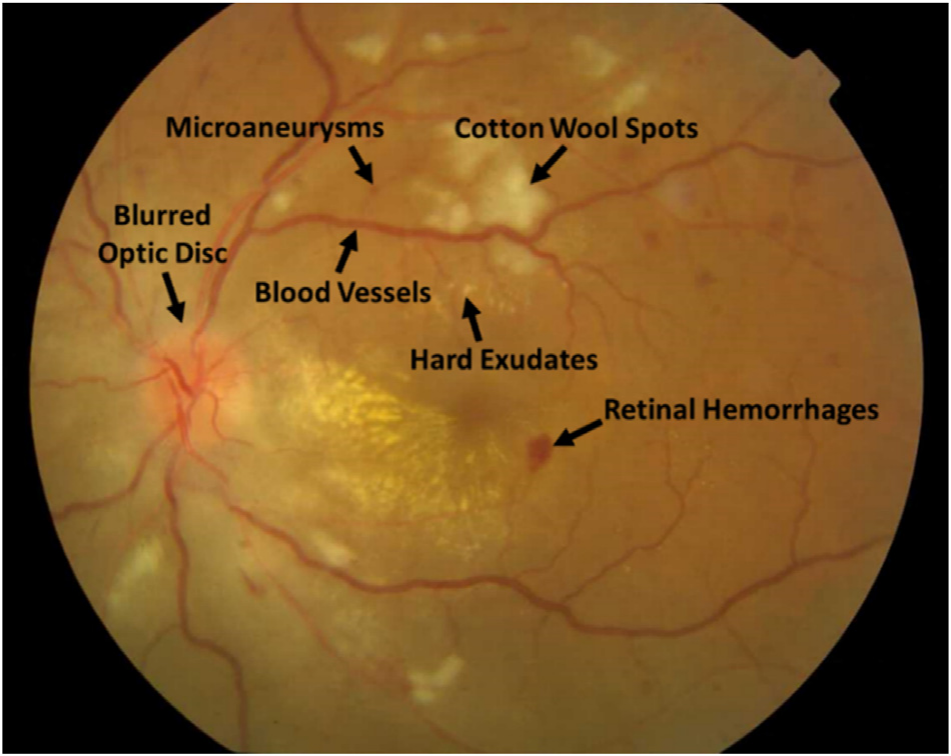
Abnormal diseased image showing various retinal abnormalities.

The dataset [1] consists of 100 fundus images (86 maculae centred and 14 optic disc centred). In maculae centred images, 76 images are diseased and 10 images are normal or healthy. In optic disc centred images, 10 images are healthy and 04 images are diseased. Out of 80 diseased images, 12 contains exudates, 07 contains haemorrhages, 04 contains papilledema, 45 contains hypertensive retinopathy signs, 08 contains cotton wool spots and 04 contains diabetic retinopathy signs. The retinal abnormalities can be tested through this dataset for automatic diagnosis. The randomly selected abnormal retinal image from the dataset is shown in Fig. 1.

Fundus retinal images are very useful to document the various retinal structures. The vascular network, optic disc, maculae, fovea and syndromes can be seen through magnified digital retinal image which is captured by using ophthalmoscope or fundus camera. The structural information of retinal image is very helpful for diagnosis and treating the various retinal diseases.

**Fig. 2:**
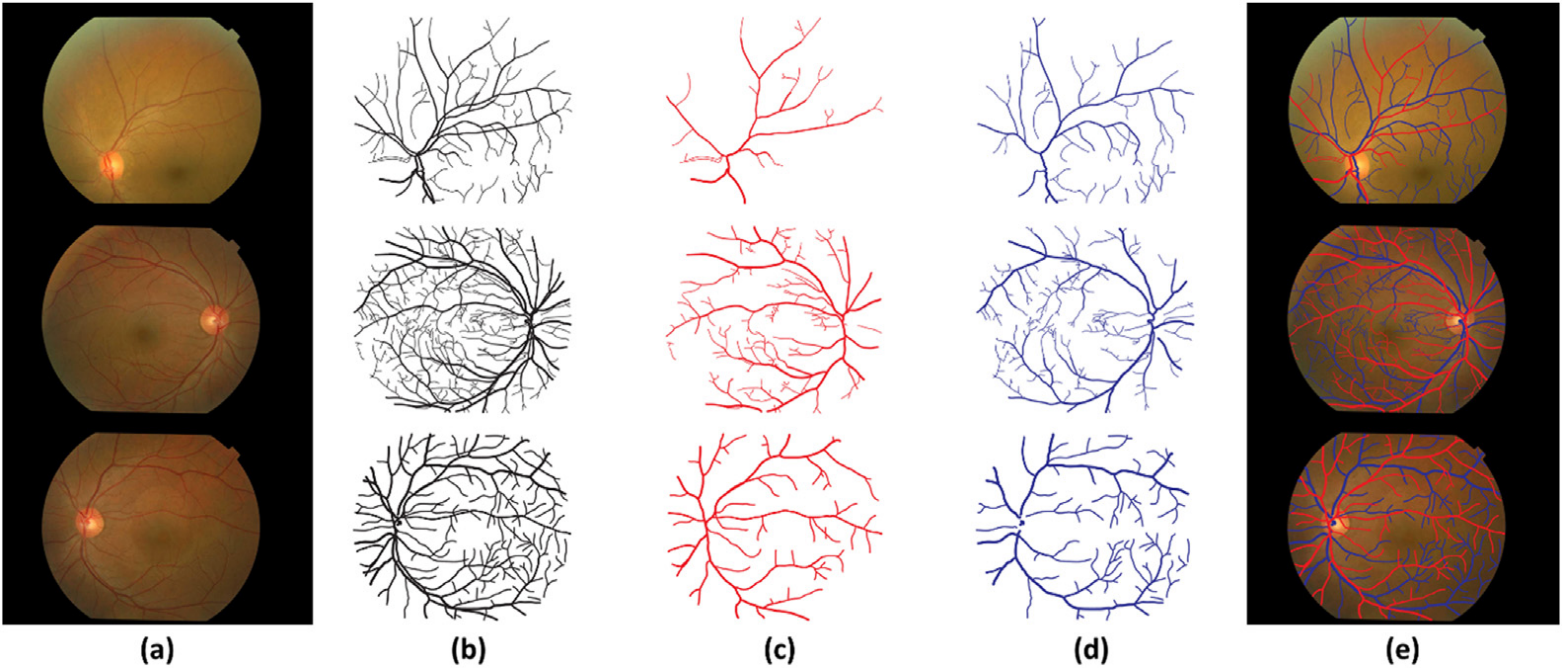
Fundus images in presented dataset, (a) original images, (b) annotated retinal blood vessels, (c) annotated retinal arteries, (d) annotated retinal veins, (e) annotated vascular networks mapped on original image.

The 100 images dataset of both eyes is annotated with the help of four experts of AFIO through Adobe Illustrator CC software. These annotations contain vascular pattern; vascular network of arteries and veins, and various pathologies. The 100 images ground truth data was also labeled by ophthalmologists of AFIO. The randomly selected three retinal images from the dataset having annotations of vascular patterns, arteriolar and venule networks with mapping of original images are shown in Fig. 2.

## 2 Experimental design, materials, and methods

The 100 images dataset is collected from ophthalmologists of AFIO. The ophthalmologists selected the retinal images of observed patients on the basis of their clinical history and medical examination. The patients having normal medical checkup results are categorized into healthy persons and their captured images are labeled as normal. The equally proportion of both male and female patients were selected of age between 25 and 80 years old. The patient's images were captured through TOPCON TRC-NW8. The centred on optic disc with resolution of 1504 x 1000 and 30-degree field of view (FOV), JPEG uncompressed images were used to build a dataset. Poor and unclear images were rejected. The dataset had been used for automated vessels segmentation; artery/vein classification; hypertensive retinopathy and papilledema detection [[2], [3], [4]].

## References

[1] Akhbar Shahzad, Hassan Taimur, Akram Muhammad Usman, Yasin Ubaidullah, Basit Imran (2017). AVRDB: Annotated dataset for vessel segmentation and calculation of arteriovenous ratio. International Conference on Image Processing, Computer Vision, and Pattern Recognition (IPCV).

[2] Akbar Shahzad, Akram Muhammad Usman, Sharif Muhammad, Tariq Anam, Khan Shoab Ahmed (2018). Decision support system for detection of hypertensive retinopathy using arteriovenous ratio. Artif. Intell. Med..

[3] Akbar Shahzad, Akram Muhammad Usman, Sharif Muhammad, Tariq Anam, Yasin Ubaidullah (2018). Arteriovenous ratio and papilledema based hybrid decision support system for detection and grading of hypertensive retinopathy. Comput. Methods Progr. Biomed..

[4] Akbar Shahzad, Akram Muhammad Usman, Sharif Muhammad, Tariq Anam, Yasin Ubaidullah (2017 Apr 1). Decision support system for detection of papilledema through fundus retinal images. J. Med. Syst..

